# Quantifying the incidence of severe-febrile-illness hospital admissions in sub-Saharan Africa

**DOI:** 10.1371/journal.pone.0220371

**Published:** 2019-07-25

**Authors:** Paul Roddy, Ursula Dalrymple, Tomas O. Jensen, Sabine Dittrich, V. Bhargavi Rao, Daniel A. Pfeffer, Katherine A. Twohig, Teri Roberts, Oscar Bernal, Ethan Guillen

**Affiliations:** 1 Médecins Sans Frontières – Febrile Illness Diagnostic Programme, New York, United States of America; 2 University of Oxford, Big Data Institute, Li Ka Shing Centre for Health Information and Discovery, Oxford, United Kingdom; 3 Foundation for Innovative New Diagnostics (FIND), Geneva, Switzerland; 4 University of Oxford – Nuffield School of Medicine, Oxford, United Kingdom; 5 Médecins Sans Frontières – Manson Unit (MSF UK), London, United Kingdom; 6 Menzies School of Health Research and Charles Darwin University, Darwin, Australia; 7 Médecins Sans Frontières – Access Campaign, Geneva, Switzerland; Lancaster University, UNITED KINGDOM

## Abstract

Severe-febrile-illness (SFI) is a common cause of morbidity and mortality across sub-Saharan Africa (SSA). The burden of SFI in SSA is currently unknown and its estimation is fraught with challenges. This is due to a lack of diagnostic capacity for SFI in SSA, and thus a dearth of baseline data on the underlying etiology of SFI cases and scant SFI-specific causative-agent prevalence data. To highlight the public health significance of SFI in SSA, we developed a Bayesian model to quantify the incidence of SFI hospital admissions in SSA. Our estimates indicate a mean population-weighted SFI-inpatient-admission incidence rate of 18.4 (6.8–31.1, 68% CrI) per 1000 people for the year 2014, across all ages within areas of SSA with stable *Plasmodium falciparum* transmission. We further estimated a total of 16,200,337 (5,993,249–27,321,779, 68% CrI) SFI hospital admissions. This analysis reveals the significant burden of SFI in hospitals in SSA, but also highlights the paucity of pathogen-specific prevalence and incidence data for SFI in SSA. Future improvements in pathogen-specific diagnostics for causative agents of SFI will increase the abundance of SFI-specific prevalence and incidence data, aid future estimations of SFI burden, and enable clinicians to identify SFI-specific pathogens, administer appropriate treatment and management, and facilitate appropriate antibiotic use.

## Introduction

Despite being a commonly documented and globally recognized cause of morbidity and mortality in communities worldwide, the global burden of severe febrile illness (SFI) is currently unknown. The inability to measure the contribution of individual fever-inducing pathogens has historically provided a major barrier to producing estimates of global SFI burden [1]. In low- and middle-income countries (LMICs) in particular, and with the exception of select syndromes (e.g. diarrhea) and diseases (e.g. malaria), there is a dearth of SFI-specific causative-agent prevalence data, owing to limited SFI diagnostic capacity [2].

Nevertheless, in sub-Saharan Africa (SSA), fever is frequently reported as a common reason for seeking healthcare [3,4]. Because many fevers in SSA are treated using both modern and traditional medicines at the community level (i.e. informal settings), SFI data from formal health facilities greatly underestimate the overall magnitude of SFI in SSA [5,6]. Moreover, there is a paucity of baseline data describing the underlying etiology of SFI cases in SSA at both community- and health facility-level [7]. Non-malarial causes of SFI may also be routinely misdiagnosed as malaria, due to exorbitant levels of malaria co-infection [8] and a common postulation that fever is conventionally caused by malaria, evident from the fact that appellations for fever and malaria have historically been used interchangeably in SSA [9]. Conceivably, up to a third of patients in SSA suffer from SFI and do not receive a correct diagnosis for their infection [7], with malaria traditionally being the *de facto* presumptive diagnosis [7]. Meanwhile, successful malaria control efforts have lowered malaria transmission in much of SSA [10] to the point where bacterial and viral pathogens now drive SFI in this region [7].

Malaria overdiagnosis remains a significant problem throughout SSA [11–20], with a considerable burden on impoverished communities [5,7,21]. Most febrile illnesses are still presumptively treated as malaria despite growing evidence that, especially in urban contexts, malaria is likely to be a declining concern [7]. Because malaria can present as SFI, malaria algorithms that are exclusively clinical (i.e. without parasite-based diagnosis) typically result in fewer true-negatives and a higher rate of false-positives [7,22,23], thus hindering differential diagnoses [7,24,25]. Numerous healthcare facilities still lack parasite-based diagnostic capacity for malaria. Although the rate of diagnostic testing in clinics is rising, from 36% of suspected malaria cases presenting to public health clinics in SSA in 2010 to 87% in 2016 [26], presumptive treatment is still common in many countries across the continent, particularly amongst individuals who seek treatment at informal health facilities [7,26–30].

Established surveillance systems conventionally report the incidence of clinical malaria based on the number of febrile individuals diagnosed with a presumed or confirmed malaria infection [8,26]. Though a high proportion of fever cases are concomitant with an RDT-confirmed *Plasmodium falciparum* malaria infection, modeling indicates that only a third of malaria-positive fevers are causally ascribed to malaria [8], and that the preponderance of febrile illness in SSA is caused by pathogens other than *P*. *falciparum*, including in locations where malaria is immensely prevalent [8]. For example, Dalrymple *et al*. [8] suggested that in a two-week period in 2014, one in four children under five years of age living in areas of stable malaria transmission were afflicted by a fever not caused by *P*. *falciparum* malaria, whereas only one in every 32 children suffered a fever causally attributable to *P*. *falciparum* [8]. Thus, SSA malaria-burden estimations based on malaria RDT-positive cases of fever are conceivably overestimating the burden by up to 67%, and this overestimation is plausibly heterogeneous between countries [8]. These results have implications for the effectiveness of treatment received at the point of care, as a substantial proportion of RDT malaria-positive fevers are likely to be asymptomatic malaria infections that are coincident with a non-malarial febrile illness (NMFI) [8]. In such instances, concomitantly treating the NMFI and the malaria infection with effective treatment is imperative. Finally, a lack of diagnostic capacity for other causative agents of SFI hinders the collection of prevalence data for SFI, which could be used to derive more accurate estimates of the burden of SFI and improve clinical algorithms.

A large proportion of NMFIs are caused by bacterial bloodstream infection and bacterial zoonoses, and both are associated with considerable mortality [1,31,32]. Bacterial bloodstream infection is a major cause of fever among hospitalized patients in SSA; in a systematic review of patients with SFI, bacteraemia was detected in 10.4% of patients in East Africa, and 12.4% of patients in West Africa [31]. Meanwhile, bacterial sepsis is a leading cause of mortality among febrile patients in LMICs, but blood culture services are not widely available, impeding pathogen-specific diagnoses [32]. A review by Reddy *et al*. (2010) determined that bacterial bloodstream infections were commonplace among hospitalized adults and children in Africa [33]. Their review confirmed that, among hospitalized patients in some locations, the prevalence of community-acquired bacteraemia surpassed that of malaria parasitaemia [33].

Severe bacterial infection is also a particular problem in neonates. Bloodstream infection, meningitis, and pneumonia are important contributors to the global burden of disease, accounting for a possible 0.68 million neonatal deaths and about 3% of all disability-adjusted life years (DALYs), a similar burden to that of HIV/AIDS [34]. Most of these DALYs are attributable to deaths because infections are a leading cause of the 2.9 million global neonatal deaths [34]. Despite robust estimates for the individual contributions of specific bacterial infections to the global burden of disease, estimates of the burden of all-cause SFI have yet to be produced.

In addition to providing essential data for robust burden estimates, it has been recognized that having accessible diagnostics is one cornerstone of a strong health care system with the ability to inform patient management in real time and influence long-term public health decision making [35]. Despite significant testing capabilities in high-income countries, diagnostic capacity in LMICs is limited, which hinders the estimation of SFI burden. Often, multiplex diagnostic tests currently on the market *i*) are restricted to molecular or immunoassay testing; *ii*) require an upfront blood culture to improve sensitivity (or other upfront unfeasible sampling procedures); *iii*) are expensive and non-robust; and *iv*) are comprised of a limited testing menu, unrepresentative of the breadth of pathogens apropos to LMICs. Hence, a number of initiatives have been launched to address this challenge, such as the scoping and preliminary development of rapid multiplex diagnostics requiring a single clinical blood specimen [36–40].

While a multitude of studies document the global breadth and diversity of causative agents of SFI, their data do not provide the necessary continuity and consistency required to quantify global or region-specific SFI burden. Thus, in this study, we aimed to quantify the incidence of SFI hospital admissions in SSA by constructing a Bayesian model dependent on a previously approximated SSA-national-level prevalence of community-acquired febrile illness [8]. The results presented herein will provide the first estimates of the burden of SFI amongst inpatient department (IPD) admissions in SSA and will inform the utility and placement of improved fever diagnostic tools for near-patient testing, which, if implemented, would improve data availability for SFI as well as treatment and patient-management strategies and clinical outcomes.

## Methods

### Hospital IPD-admission data for SFI

To inform our herein described Bayesian model, a dataset of total hospital IPD admissions for SFI in SSA countries was constructed. Data were sought for each SSA country with annual IPD admission reports obtainable through two separate search strategies. First, an initial Google search was conducted using the terms “Ministry of Health”, the name of the country, and “malaria”. A second round of searches was then performed for each SSA country using context-specific terms (e.g. the official name of the national health or statistics authority and/or the title of country-specific health bulletins or reports). The term “malaria” was included as only geographic areas of SSA countries with stable *P*. *falciparum* transmission were incorporated into our model.

Search results yielded, from 2006 to 2014 and from 12 SSA countries, 45 annual reports detailing IPD admissions for all ages (Table 1). The reports were either annual Ministry of Health (MoH) reports or official annual health statistics presented and released by the relevant national authority for each identified country. Report references are provided in S1 Table.

**Table 1:** List of SSA annual-country-reports of IPD admissions employed for our analyses. For each country-year, contemporaneous population projections from the United Nations [42] were applied to calculate SFI IPD admissions per 1000 population. The prevalence of community-acquired fever (of any severity and any cause) was extracted from Dalrymple *et al*. [8]. Report reference identification numbers are listed in S1 Table.

Country	Year	Population (UN)	SFI IPD Admissions (Total)	SFI IPD Admissions (per 1000)	Prevalence of fever acquired in the community (Dalrymple *et al*.)	Report ID
Burkina Faso	2006	13,834,195	190,967	13.8	0.2570	1
Burkina Faso	2007	14,264,002	259,179	18.2	0.2673	2
Burkina Faso	2008	14,709,011	462,282	31.4	0.2594	3
Burkina Faso	2009	15,165,856	374,758	24.7	0.2716	4
Burkina Faso	2010	15,632,066	467,895	29.9	0.2440	5
Burkina Faso	2011	16,106,851	147,186	9.1	0.2653	6
Burkina Faso	2012	16,590,813	618,061	37.3	0.3438	7
Burkina Faso	2013	17,084,554	156,648	9.2	0.4195	8
Burkina Faso	2014	17,589,198	289,907	16.5	0.4209	9
Burundi	2010	9,461,117	252,150	26.7	0.2278	10
Burundi	2011	9,790,151	314,089	32.1	0.3799	11
Burundi	2012	10,124,572	327,454	32.3	0.4415	12
Burundi	2013	10,465,959	430,284	41.1	0.4112	13
Burundi	2014	10,816,860	747,262	69.1	0.3432	14
Chad	2006	10,423,616	90,740	8.7	0.2990	15
Chad	2012	12,715,465	35,483	2.8	0.3485	16
Chad	2013	13,145,788	165,135	12.6	0.3758	17
eSwatini	2011	1,212,458	15,217	12.6	0.1033	36
Gambia	2006	1,487,731	19,210	12.9	0.2057	18
Gambia	2007	1,536,424	20,424	13.3	0.2297	19
Gambia	2008	1,586,749	17,948	11.3	0.2301	20
Gambia	2009	1,638,899	46,139	28.2	0.1650	21
Gambia	2011	1,749,099	30,797	17.6	0.1994	22
Gambia	2013	1,866,878	24,745	13.3	0.1575	23
Liberia	2014	4,396,554	73,119	16.6	0.4255	24
Madagascar	2006	18,826,129	64,363	3.4	0.2670	25
Madagascar	2007	19,371,031	20,373	1.1	0.2659	26
Madagascar	2008	19,926,798	49,689	2.5	0.2231	27
Madagascar	2009	20,495,706	39,854	1.9	0.1814	28
Madagascar	2010	21,079,532	42,227	2	0.1516	29
Madagascar	2011	21,678,867	39,209	1.8	0.1611	30
Madagascar	2013	22,924,557	62,058	2.7	0.1295	31
Madagascar	2014	23,571,713	54,167	2.3	0.1784	32
Rwanda	2011	10,556,429	179,860	17	0.1549	33
Rwanda	2012	10,817,350	158,535	14.7	0.1494	34
Rwanda	2013	11,078,095	113,113	10.2	0.1852	35
Tanzania	2012	48,645,709	535,530	11	0.2265	37
Togo	2015	7,304,578	51,887	7.1	0.2663	38
Uganda	2015	39,032,383	613,352	15.7	0.3646	39
Zimbabwe	2009	13,720,997	92,639	6.8	0.2202	40
Zimbabwe	2010	13,973,897	144,963	10.4	0.1839	41
Zimbabwe	2011	14,255,592	73,468	5.2	0.1792	42
Zimbabwe	2012	14,565,482	129,244	8.9	0.1412	43
Zimbabwe	2013	14,898,092	43,129	2.9	0.1699	44
Zimbabwe	2014	15,245,855	99,058	6.5	0.1733	45

In each report, a primary cause for all IPD admissions was presented thereby allowing the total IPD admissions for clinical presentations that are associated with SFI to be summed and extracted (denoted as “SFI IPD Admissions” in Table 1). ‘Severity’ was defined as admission to an IPD only and was not based on specific severity signs. ‘Febrile’ was defined as having a recorded primary diagnosis on IPD admission with an illness capable of producing fever and was not based on a body-temperature measurement. To account for health-facility IPD admissions not captured by the respective national Health Management Information System (HMIS) in each SSA country, a reporting completeness adjustment was applied. For 17 reports, the total SFI-IPD cases summed from each report was adjusted according to the report’s stated rate of national reporting completeness. For the remaining 28 reports, where reporting completeness was not stated, reporting coverage was imputed from the 2017 WHO World Malaria Report [26], which documents countries’ current and past health system reporting completeness. Finally, to calculate incidence rates for SFI-IPD admissions, each of the 45 reports was matched to contemporaneous population projections generated by the United Nations (UN) [41].

### National prevalence of community-acquired febrile illness

Next, national-level modeled estimates of community-acquired febrile-illness prevalence amongst children under five years of age were procured from a previous publication [8] and matched to each country-year with an obtained report. These prevalence estimates were produced using a Bayesian hierarchical modeling approach. In this approach, household survey data on the prevalence of fever (amongst children under five years of age in the two weeks preceding each survey) was used in conjunction with remotely-sensed environmental and socioeconomic variables to predict continental-scale cartographic estimates of the prevalence of community-acquired febrile illness for children under five years of age across all geographic locations in SSA with stable *P*. *falciparum* transmission at a 5km by 5km spatial resolution [8].

Although the community-acquired febrile-illness prevalence estimates from the aforementioned publication [8] were restricted to children under five years of age, our Bayesian model described below assumed they were informative of the relative prevalence of community-acquired febrile-illness in individuals of all ages. Specifically, from the procured estimates [8], the following were extracted and extended to all ages: *i)* the contemporaneous prevalence of community-acquired febrile-illness for each matched country-year from our 45 obtained reports (Table 1); and *ii)* the 2014 prevalence of community-acquired febrile-illness to generate 2014 estimates of SFI-IPD admissions for all SSA countries within areas of stable *P*. *falciparum* transmission, described in detail in the subsequent subsection.

### Bayesian model

Our Bayesian model was constructed to estimate the relationship between the incidence of SFI-IPD admissions as determined by the 45 obtained reports, and the underlying prevalence of community-acquired febrile illness as previously approximated [8]. To learn this relationship, the paired observations of incidence of SFI-IPD admissions and matched national-level prevalence of community-acquired febrile illness were applied, for each of the 45 country-years, to an assumed linear correlation:

$$I_{{admission}_{i}}=(p_{{fev}_{i}}\times \alpha )+\beta$$

For each country-year, $I_{{admission}_{i}}$ is the incidence of SFI-IPD admissions (all ages) in country-year *i*, and $p_{{fev}_{i}}$ is the prevalence of community-acquired fever amongst children under five years of age in country-year *i*. The model was constructed using the Template Model Builder (TMB) package in the R programming language [42] and used a numerical optimization function to learn the gradient (*α*) and the intercept (*β*). An additional noise parameter (σ) was learned to generate mock data samples, which were used to obtain credible intervals for the relationship between *I*_*admission*_ and *p*_*fev*_.

Using our fitted model in conjunction with 2014 estimates for prevalence of community-acquired febrile illness in all SSA countries, incidence estimates of SFI-IPD admissions amongst individuals of all ages were generated. The total number of SFI-IPD admissions was subsequently calculated by multiplying the estimated incidence rate by the relevant UN contemporaneous population projections [41]. As estimates were only valid for geographic areas with stable *P*. *falciparum* transmission, the total number of SFI-IPD admissions was restricted to only stable zones of *P*. *falciparum* transmission as defined by the Malaria Atlas Project [43,44] in SSA countries with intermittent *P*. *falciparum*-transmission geography.

### Separating SFI-IPD admissions by severe malaria versus other causes

To further refine the interpretation of our findings, SFI-IPD admissions were stratified by ‘severe malaria’ versus ‘other causes’. Uncomplicated malaria was included in ‘other causes’ of SFI, motivated by the rationale that severe and uncomplicated malaria cases should be segregated, and that often uncomplicated malaria cases are coincident with an undiagnosed NMFI that is the actual underlying cause of the fever [1,8,45]. Thus, in the reviewed IPD admission reports, uncomplicated malaria was recorded but this does not necessarily indicate that it was the primary cause of illness and decision for IPD admittance. IPD admission rates for severe malaria were extracted from modeled estimates produced by Camponovo *et al*. [46].

## Results

**Fig 1:**
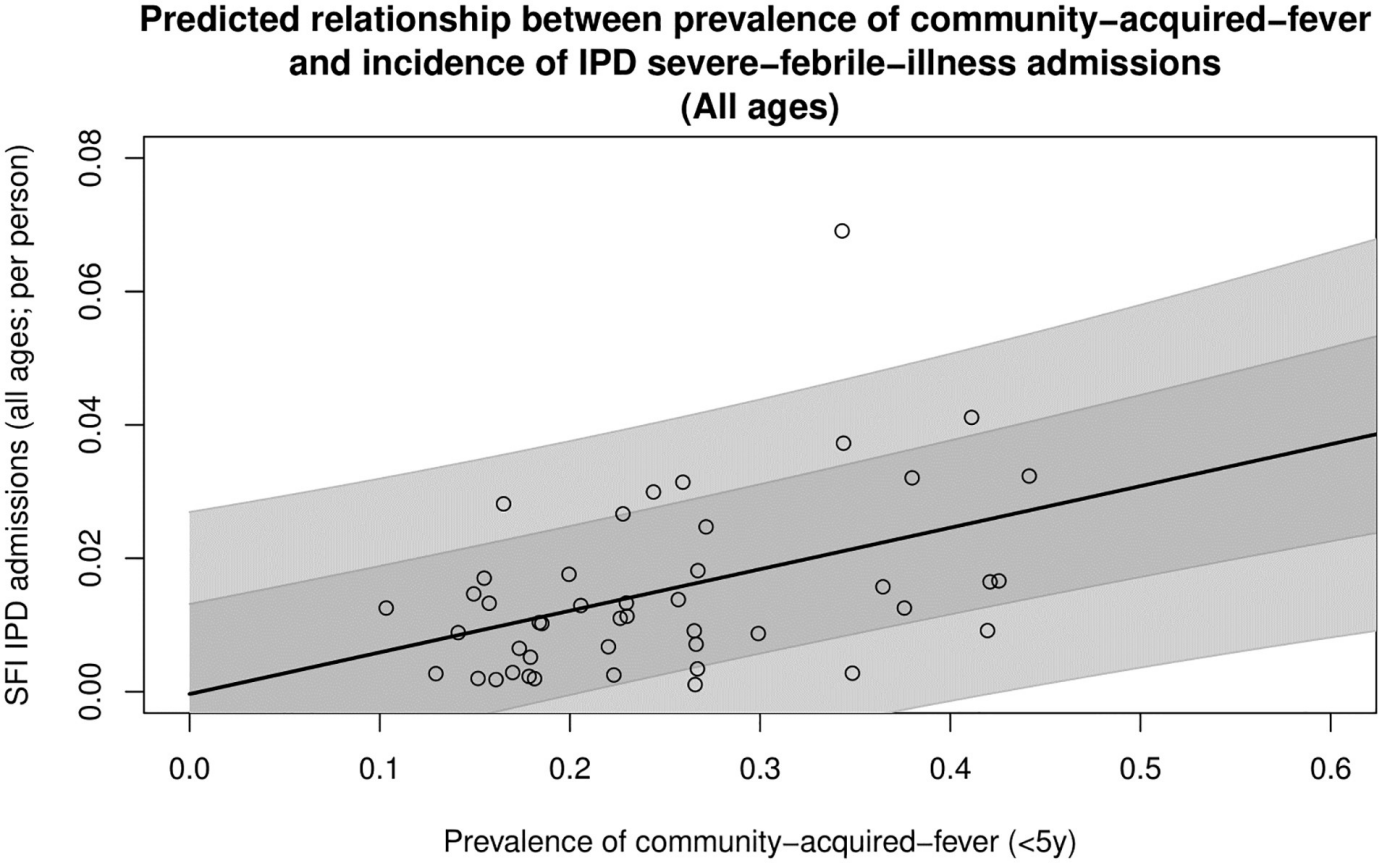
Final fitted relationship between incidence of IPD admissions for severe-febrile-illness (all ages) and estimated prevalence of community-acquired febrile illness (<5 years of age). The median of the final fitted relationship is represented by the black line, with 68% and 95% credible intervals overlain in increasingly light grey. The overlain points are the observations of the 45 country-years of the incidence of severe-febrile-illness (all ages) versus the national estimated prevalence of community-acquired fever (<5 years of age).

**Fig 2:**
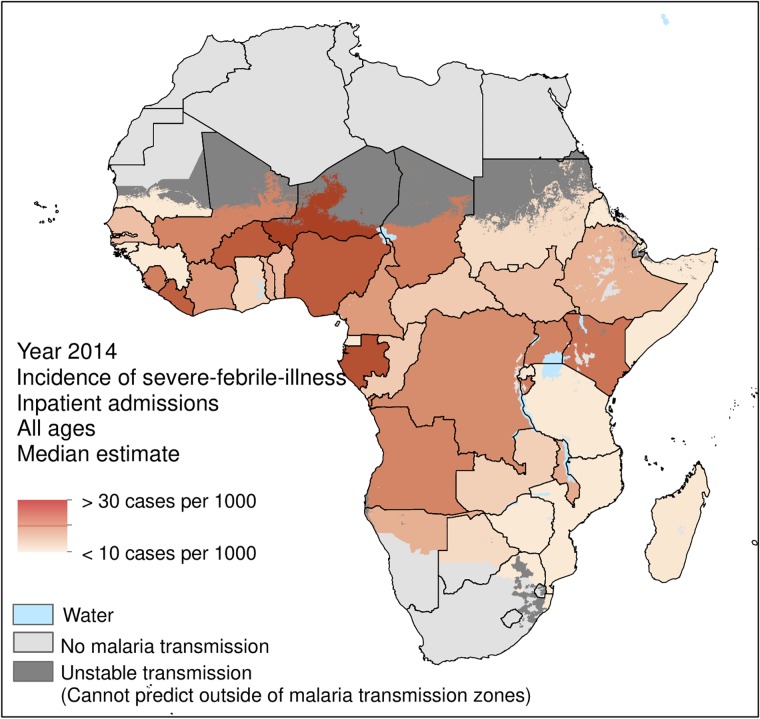
Incidence rate (per 1000 people) of SFI-IPD admissions for severe-febrile-illness in 2014 for each sub-Saharan African country within areas of stable *P*. *falciparum* malaria transmission.

The gradient (*α*) intercept (*β*), and noise parameter (σ) were optimized, and the final fitted values for each parameter is provided in Table 2. The final fitted relationship is shown in Fig 1, with the median estimate and 68% and 95% credible intervals overlain with the original 45 reported observations from which the relationship was fitted. The fitted relationship was used to generate 2014 estimates of the incidence and total SFI-IPD admissions for all SSA countries within areas of stable *P*. *falciparum* transmission (Table 3). Fig 2 presents the mapped incidence rates (per 1000 people) of 2014 SFI-IPD admissions for individuals of all ages within stable *P*. *falciparum* transmission zones of SSA. The mean population-weighted incidence of SFI-IPD admissions across SSA was 18.4 (6.8–31.1, 68% CrI) per 1000 people. The countries with the lowest incidence rates were eSwatini, Eritrea and Somalia with 1.1 (0–13.9, 68% CrI), 1.8 (0–13.9, 68% CrI), and 5.8 (0–19.1, 68% CrI) SFI-IPD admissions per 1000 people, respectively. The countries with the highest incidence rates were Niger, Gabon, and Nigeria, with 33.6 (20.6–47.8, 68% CrI), 27.7 (14.0–40.7, 68% CrI), and 26.4 (14.1–38.8, 68% CrI) SFI-IPD admissions per 1000 people, respectively. Within stable *P*. *falciparum* transmission zones of SSA, there was an estimated median total of 16,200,337 SFI-IPD admissions in 2014.

**Table 2:** Final fitted values for each of the three optimized parameters: Gradient (*α*), intercept (*β*), and noise (σ).

Parameter	*α*	*β*	σ
**Value**	0.063	-0.001	0.012

**Table 3:** Estimated 2014 incidence and total IPD admissions for SFI for each SSA country. Incidence and total SFI-IPD admissions are further stratified by severe malaria IPD admissions and IPD admissions for SFI other than severe malaria (but including uncomplicated malaria). Estimates for severe malaria IPD admissions were extracted from Camponovo *et al*. [4,6].

**PREDICTED SFI IPD ADMISSIONS—YEAR 2014**			**SFI IPD Admissions (per 1000 people)**					**SFI IPD Admissions Total**					**Severe malaria admissions (Camponovo *et al*.)**	**SFI admissions other than severe malaria**
**Sub-Saharan African Country**	**All-cause fever prevalence**	**Population (all ages; malaria zones only)**	**Low95**	**Low68**	**Median**	**Up68**	**Up95**	**Low95**	**Low68**	**Median**	**Up68**	**Up95**	**Median**	**Median**
**Angola**	0.356	24,410,939	0.0	8.8	21.1	34.9	48.3	0	213,774	515,050	852,829	1,178,733	72,281	442,770
**Benin**	0.255	10,629,584	0.0	3.5	15.2	27.5	38.7	0	36,747	161,452	292,131	411,450	23,959	137,493
**Botswana**	0.183	1,123,305	0.0	0.0	11.5	24.5	36.6	0	0	12,881	27,516	41,142	134	12,747
**Burkina Faso**	0.421	17,572,785	0.0	13.8	26.2	38.6	51.8	0	242,396	461,151	678,389	909,969	75,071	386,080
**Burundi**	0.343	9,817,194	0.0	9.1	22.1	33.4	46.8	0	88,913	217,246	327,949	459,161	33,133	184,113
**Cameroon**	0.300	22,753,850	0.0	5.8	18.6	30.5	44.1	0	132,630	423,008	694,093	1,004,250	54,632	368,376
**Central African Republic**	0.232	4,808,806	0.0	0.6	13.2	26.4	38.0	0	2,982	63,259	126,829	182,675	8,074	55,185
**Chad**	0.359	13,118,333	0.0	9.1	22.1	33.3	48.1	0	119,457	289,668	437,210	630,393	19,179	270,489
**Congo**	0.223	4,513,705	0.0	1.1	13.2	25.4	38.1	0	5,135	59,547	114,540	172,150	3,381	56,166
**Cote d’Ivoire**	0.311	22,143,458	0.0	6.2	19.4	31.9	42.1	0	138,392	428,909	705,858	931,655	27,480	401,429
**Democratic Republic of the Congo**	0.334	72,841,230	0.0	8.3	20.3	33.0	46.1	0	601,179	1,477,170	2,401,747	3,356,346	316,932	1,160,238
**Djibouti**	0.163	111,209	0.0	0.0	9.8	23.0	34.6	0	0	1,087	2,556	3,845	42	1,046
**Equatorial Guinea**	0.138	800,427	0.0	0.0	6.8	20.3	33.1	0	0	5,430	16,221	26,485	Unknown	Unknown
**Eritrea**	0.053	4,912,138	0.0	0.0	1.8	13.9	29.2	0	0	9,032	68,492	143,511	157	8,875
**eSwatini**	0.046	26,047	0.0	0.0	1.1	13.9	27.1	0	0	30	363	706	Unknown	Unknown
**Ethiopia**	0.257	78,290,677	0.0	3.2	16.0	28.2	41.0	0	254,115	1,256,453	2,205,039	3,208,625	1,801	1,254,652
**Gabon**	0.449	1,688,464	1.6	14.0	27.7	40.7	54.1	2,643	23,716	46,837	68,682	91,369	2,045	44,793
**Gambia**	0.209	1,937,715	0.0	0.0	12.6	24.5	39.8	0	0	24,346	47,560	77,092	1,895	22,451
**Ghana**	0.219	26,780,858	0.0	0.3	12.2	25.0	38.2	0	7,962	326,636	670,591	1,022,757	28,575	298,061
**Guinea**	0.160	12,224,764	0.0	0.0	8.7	22.2	34.6	0	0	105,835	271,723	423,040	14,132	91,704
**Guinea-Bissau**	0.162	1,802,157	0.0	0.0	8.9	21.5	33.7	0	0	15,987	38,773	60,728	4,172	11,815
**Kenya**	0.395	36,672,739	0.0	11.6	23.0	36.6	49.9	0	424,460	843,471	1,343,970	1,830,769	4,804	838,667
**Liberia**	0.426	4,397,036	0.8	13.5	25.8	39.2	52.8	3,423	59,370	113,398	172,435	232,129	30,643	82,755
**Madagascar**	0.178	22,809,791	0.0	0.0	10.2	22.7	37.7	0	0	233,555	518,325	859,074	6,022	227,533
**Malawi**	0.307	16,686,966	0.0	5.6	16.8	30.2	43.8	0	94,224	280,030	503,963	730,963	56,602	223,427
**Mali**	0.342	16,737,709	0.0	9.4	21.1	33.7	47.8	0	157,333	353,437	564,757	799,241	30,078	323,360
**Mauritania**	0.181	2,970,225	0.0	0.0	10.1	22.7	35.6	0	0	30,020	67,287	105,785	160	29,860
**PREDICTED SFI IPD ADMISSIONS—YEAR 2014**			**SFI IPD Admissions (per 1000 people)**					**SFI IPD Admissions Total**					**Severe malaria admissions (Camponovo *et al*.)**	**SFI admissions other than severe malaria**
**Sub-Saharan African Country**	**All-cause fever prevalence**	**Population (all ages; malaria zones only)**	**Low95**	**Low68**	**Median**	**Up68**	**Up95**	**Low95**	**Low68**	**Median**	**Up68**	**Up95**	**Median**	**Median**
**Mozambique**	0.179	27,227,056	0.0	0.0	10.1	22.9	34.7	0	0	274,814	624,190	944,165	42,556	232,259
**Namibia**	0.262	1,527,488	0.0	3.7	16.2	29.2	41.1	0	5,728	24,698	44,619	62,743	472	24,226
**Niger**	0.534	18,628,166	7.3	20.6	33.6	47.8	58.8	135,861	383,374	626,126	890,439	1,095,286	33,642	592,484
**Nigeria**	0.428	177,616,139	2.0	14.1	26.4	38.8	52.8	350,397	2,512,719	4,683,544	6,895,452	9,386,114	79,572	4,603,972
**Rwanda**	0.188	9,073,630	0.0	0.0	10.2	22.6	35.8	0	0	92,299	205,469	324,729	4,800	87,499
**Senegal**	0.255	14,680,202	0.0	2.5	14.6	26.8	38.4	0	37,254	214,317	392,805	564,166	5,652	208,665
**Sierra Leone**	0.348	6,324,359	0.0	8.5	21.7	33.6	45.8	0	53,753	137,370	212,209	289,930	39,085	98,286
**Somalia**	0.101	10,462,327	0.0	0.0	5.8	19.1	31.7	0	0	60,982	199,364	331,928	157	60,826
**South Africa**	0.129	6,324,835	0.0	0.0	7.6	20.2	31.1	0	0	48,069	127,669	197,003	Unknown	Unknown
**South Sudan**	0.254	11,843,726	0.0	3.8	14.7	28.0	43.1	0	45,542	174,692	331,612	510,919	Unknown	Unknown
**Sudan**	0.201	36,893,859	0.0	0.0	11.5	24.4	39.0	0	0	422,952	901,260	1,437,031	8,486	414,467
**Togo**	0.266	7,112,093	0.0	3.4	16.0	29.6	43.0	0	24,111	113,825	210,496	305,786	15,981	97,844
**Uganda**	0.365	36,946,132	0.0	8.9	21.4	34.3	47.0	0	327,983	789,580	1,268,463	1,736,127	76,589	712,991
**United Republic of Tanzania**	0.148	50,854,889	0.0	0.0	8.6	20.8	34.0	0	0	438,610	1,059,202	1,730,260	64,281	374,330
**Zambia**	0.200	15,755,833	0.0	0.0	12.6	25.7	39.4	0	0	198,919	404,207	621,504	42,809	156,110
**Zimbabwe**	0.173	15,113,578	0.0	0.0	9.6	22.1	34.1	0	0	144,609	334,496	515,777	4,549	140,060
**TOTAL**	-	**878,966,426**	**0.6**	**6.8**	**18.4**	**31.1**	**44.3**	**492,324**	**5,993,249**	**16,200,337**	**27,321,779**	**38,947,507**	**1,234,013**	**14,738,103**

**Fig 3:**
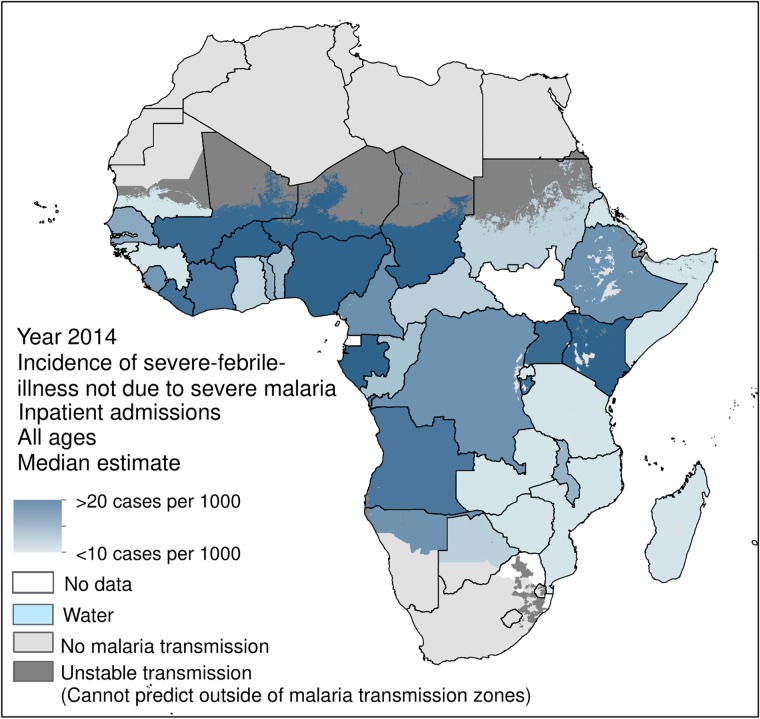
Incidence rate (per 1000 people) of 2014 IPD admissions for severe-febrile-illness due to all illnesses other than severe malaria (but including admissions for uncomplicated malaria) for each sub-Saharan African country within areas of stable *P*. *falciparum* transmission. Estimates for severe malaria IPD admissions were unavailable for Equatorial Guinea, South Africa, South Sudan, and eSwatini, thus these countries were not included in this section of the analysis.

**Fig 4:**
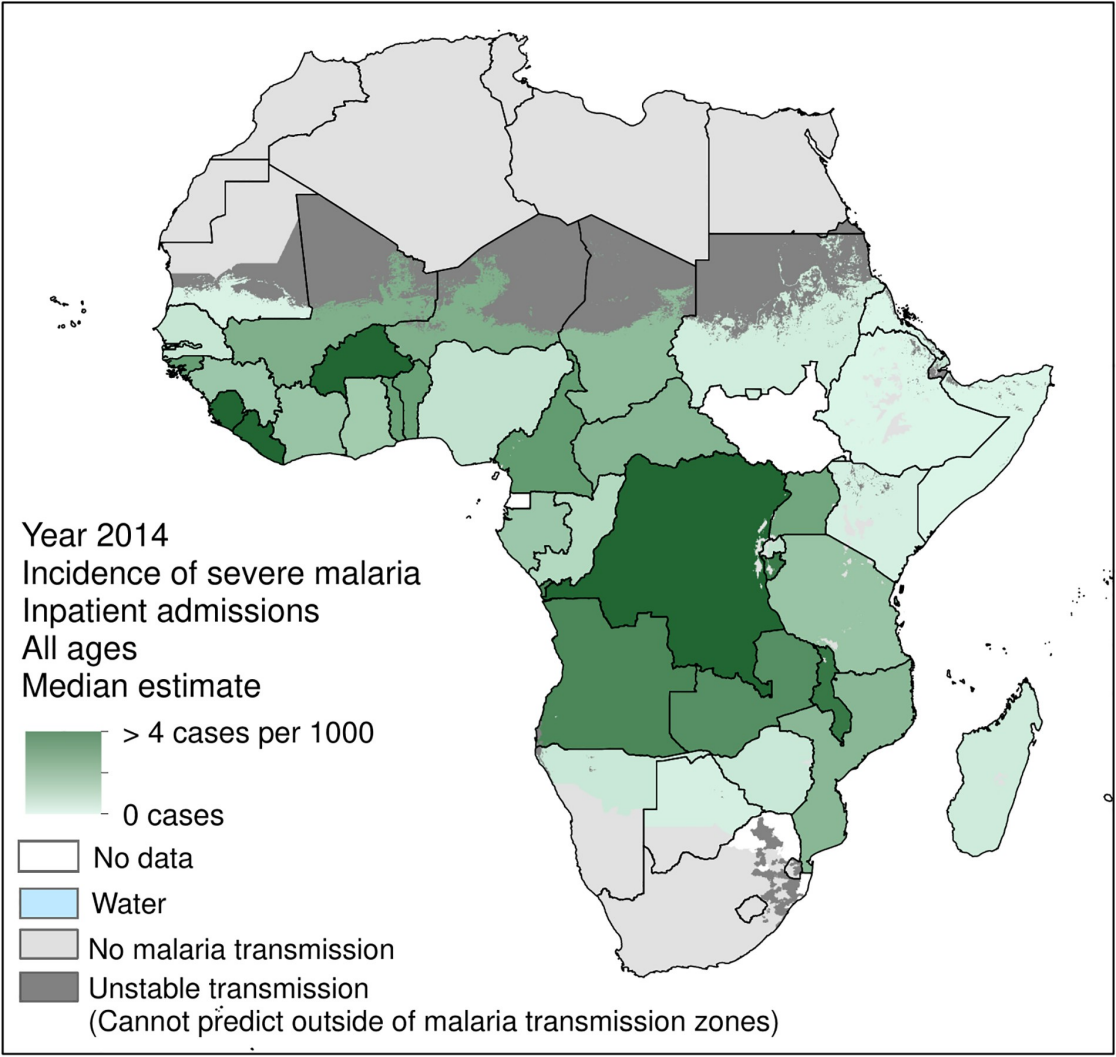
Incidence rate (per 1000 people) of 2014 IPD admissions for severe malaria (not including uncomplicated malaria) for each sub-Saharan African country within areas of stable *P*. *falciparum* malaria transmission. These estimates were produced by Camponovo *et al*. [46]. Estimates for severe malaria IPD admissions were unavailable for Equatorial Guinea, South Africa, South Sudan, and eSwatini, thus these countries were not included in this section of the analysis.

When SFI-IPD admissions due to severe malaria were separated and removed, the 2014 SFI-IPD population-weighted incidence rate, including uncomplicated malaria cases, fell to 16.8 per 1000 people (Fig 3). A corollary of this was that the 2014 population-weighted incidence rate of severe-malaria-SFI-IPD admissions was 1.4 per 1000 people. Fig 4 maps the 2014 incidence rates of severe-malaria-SFI-IPD admissions for each SSA country, as detailed in Camponovo *et al*. [46].

**Fig 5:**
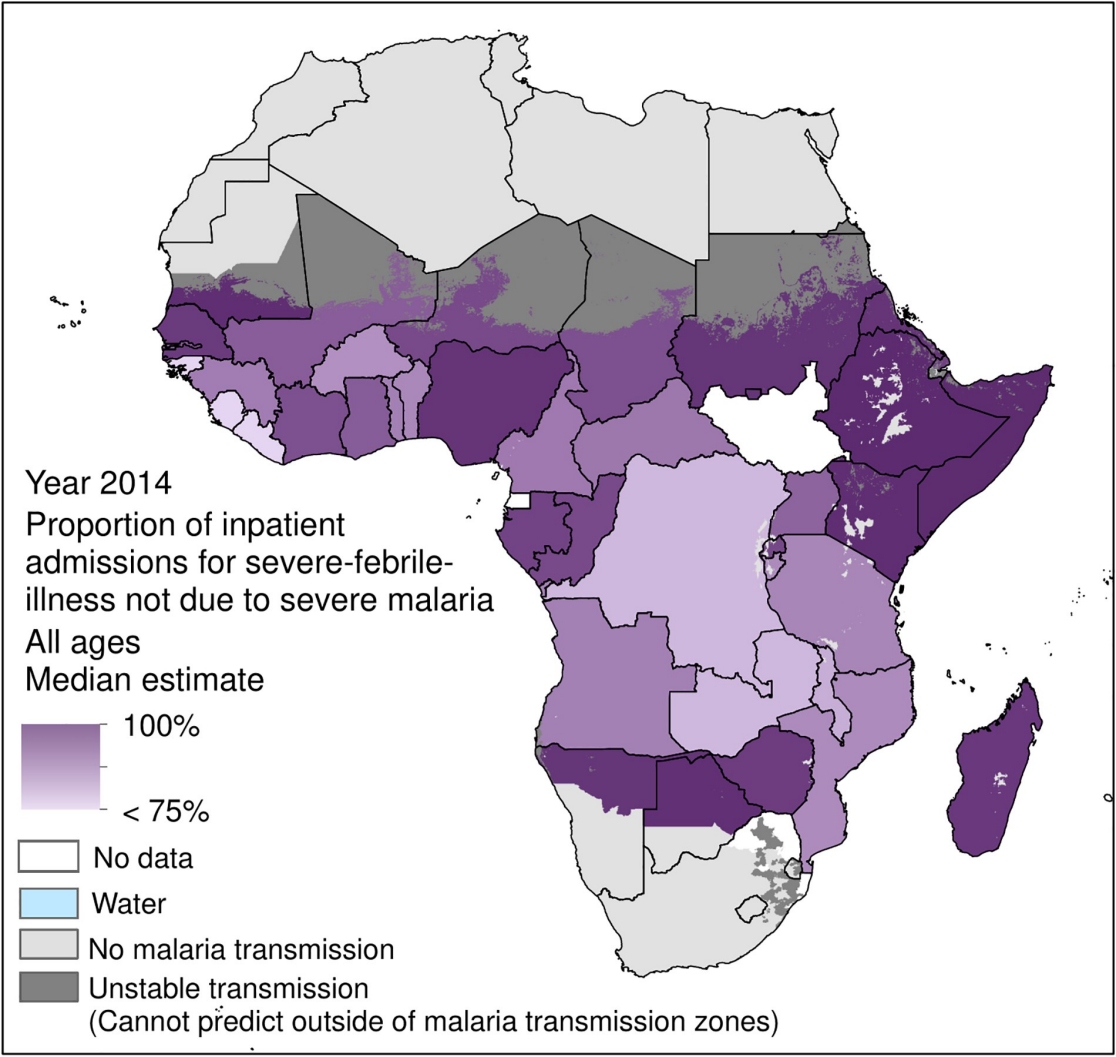
The proportion of 2014 IPD admissions for severe-febrile-illness that were causally due to illnesses other than severe malaria for each sub-Saharan African country within areas of stable *P*. *falciparum* transmission. Illnesses other than severe malaria include admissions for uncomplicated malaria. Estimates for severe malaria IPD admissions were unavailable for Equatorial Guinea, South Africa, South Sudan, and eSwatini, thus these countries were not included in this section of the analysis.

Fig 5 presents the proportion of 2014 SFI-IPD admissions that were causally due to illnesses other than severe malaria. Within stable *P*. *falciparum* transmission zones of SSA, the 2014 population-weighted proportion of SFI-IPD admissions due to illnesses other than severe malaria (but including uncomplicated malaria) was 84.1%. The SSA countries with the lowest 2014 proportion of SFI-IPD admissions due to illnesses other than severe malaria (but including uncomplicated malaria) were Sierra Leone, Liberia, and Guinea-Bissau (71.5%, 73.0%, and 73.9% respectively) while the SSA countries with the highest 2014 proportion were Ethiopia, Somalia and Mauritania (99.9%, 99.7%, and 99.5% respectively). Estimates of severe malaria SFI-IPD admissions were not available for Equatorial Guinea, South Africa, South Sudan, and eSwatini as the publication by Camponovo *et al*. did not include estimates for those countries [46]. Thus, as these four countries did not contribute towards continental-SSA population-weighted estimates, comparative analyses of SFI-IPD admissions due to severe malaria versus other causes could not be enumerated.

## Discussion

This study quantified the incidence of SFI hospital admissions in SSA with the aim of increasing the understanding of SFI burden in SSA, through estimation of the incidence and total 2014 SFI-IPD admissions for SSA countries within areas of stable *P*. *falciparum* transmission. The 2014 mean population-weighted SFI-IPD-admission incidence rate across SSA for all ages was 18.4 (6.8–31.1, 68% CrI) per 1000 people; when SFI-IPD admissions due to severe malaria were separated and removed, the 2014 population-weighted SFI-IPD-admission incidence rate fell to 16.8 per 1000 people, suggesting only 8.7% of SFI-IPD-admissions in SSA in 2014 were due to severe malaria, though its magnitude of variation by country ranged from 28.5% of all SFI-IPD admissions in Sierra Leone to only 0.1% in Ethiopia.

Our estimates suggest that SFI is a significant public health burden in SSA, particularly given that only estimates for IPD admissions are presented, and the community burden of SFI without inpatient care is likely to be significantly higher. Given the limited diagnostic capacity in LMICs for most non-malarial SFI, our study suggests that, among other interventions, clinicians in SSA would benefit from increased diagnostic facilities in order to appropriately treat infections and have an increased clinical awareness of the high incidence of non-severe-malarial SFI.

Estimates of SFI-IPD admissions in SSA were hitherto unknown and yet are crucial when deciding on new intervention strategies. The findings presented here identify the need for new routine diagnostic tools for SFI, which, in addition to improving patient outcomes, can inform SFI intervention strategies. As well as providing insights into the burden of SFI in hospitals across SSA, this analysis was designed to gauge the addressable market in SSA for potential new diagnostic innovations, such as a multiplex and multianalyte test for multiple pathogens and analyte types [35].

While presenting a unique view of the SFI burden with novel data for SSA, our study had a significant number of limitations. First, it is paramount to emphasize that our Bayesian model was not designed to identify and analyze specific patient-subgroups, such as neonates or those with an advanced HIV infection, who are likely to have an elevated incidence of SFI-IPD admissions compared to a general SFI-patient-population presenting to an IPD in a LMIC. The model did not take into consideration that SSA countries have varying IPD-admission criteria and bed capacity. Hence, a patient with SFI of unknown etiology could be treated in an outpatient department in one location while, in another, treated in an IPD and thereby meet our definition for SFI. In addition, in some locations, some SFI patients requiring IPD admission could be refused admission due to bed-capacity limitations. Therefore, we underscore that our estimates predominantly reflect the number of SFI-IPD admissions and not the incidence of SFI that require an IPD admission. Finally, our modeling approach could not account for seasonal fluctuations in SFI-IPD admissions, which may be programmatically relevant for intervention strategies.

Serving to construct our dataset of total SFI-IPD admissions in SSA countries and subsequently inform our Bayesian model, the 45 obtained MoH annual reports detailing IPD admissions for all ages were available from 2006 to 2014 only, thus our estimates are not necessarily applicable to the present. The 45 included reports were collated from 12 SSA countries only, and therefore not necessarily representative of the other SSA countries they were extrapolated to. Some countries (Burkina Faso, Burundi, Chad, Gambia, Madagascar, Rwanda, and Zimbabwe) provided multiple reports over the study period, so these countries exerted a higher level of influence over the final model fit than countries where only one report was available during the study period. The limited geographical coverage of the included reports contributed towards large levels of uncertainty associated with our estimates, as detailed in Table 3. Additionally, as not all IPDs report their data to their respective national HMIS, the reported SFI-IPD admissions were extracted from the 45 national reports and adjusted according to the report’s stated rate of national reporting completeness, where available (17 reports). For the remaining 28 reports without a stated rate of national reporting completeness, SFI-IPD admissions were adjusted by imputing reporting coverage from an alternative health-system-reporting-completeness source, the 2017 WHO World Malaria Report [26]. Thus, the robustness of these adjustment rates is uncertain.

Reports were also inconsistent in their selection and reporting of illnesses, exemplified by the definitions for severity and fever being dependent on the subjective recordings and decisions of IPD staff and not actual signs of severity or elevated and measured body temperatures. Thus, some SFI in our dataset could have presented without fever as, for example, acute gastroenteritis can result in severe dehydration requiring IPD admission, but fever is often not present, though this condition was recorded as SFI as fever can be present on some occasions. We included all febrile illnesses regardless of their routine method of detection; therefore, researchers using our estimates to guide the development of diagnostics requiring a whole blood sample should be aware that the estimates presented here include SFI from illnesses such as urinary tract infections and other conditions that are usually not accompanied by blood stream invasion, and may not be diagnosable via such tests. Further, our estimates pertain to all SFI-IPD admissions and do not estimate the incidence and number of SFI admissions that are of an unknown source, which researchers using the results presented here should also be cognizant of. This inconsistency in SFI definitions between reports introduced a significant amount of noise into the fitted relationship between community fever prevalence and SFI IPD admissions (Fig 1), which is reflected in the final uncertainties associated with our estimates (Table 3).

There was a multitude of caveats with our community-acquired febrile-illness prevalence estimates from our applied source [8]. The reported prevalence estimates were amongst children under 5 years of age only whereas the 45 annual reports detailing IPD admissions were from all ages. As no published scientific studies were available to confirm a commensurate relationship, we assumed that the relative prevalence of community-acquired febrile-illness in children under 5 years of age was applicable to individuals of all ages, whereby an area of high community-acquired febrile-illness prevalence in children will equate to a high prevalence in other age groups. Our predication, if erroneous, would undermine our model’s estimations, likely through overestimation. Secondly, the study’s household survey data detailed self-reported fevers of any severity over the previous two weeks, which contrasts with the 45 reports that captured individuals who had access to and sought IPD care for their fever-inducing illness. This means that an unknown proportion of non-severe fevers likely not requiring IPD hospitalization contributed an unknown degree of imprecision to our continent-wide incidence estimates. Third, the study was geographically limited to areas of SSA with stable *P*. *falciparum* transmission thereby confining our Bayesian hierarchical modeling approach to a commensurate geography.

Our modelling approach did not assess potential confounders of the relationship between SFI-IPD admissions and community-acquired febrile-illness prevalence. Two confounders may lead to the relationship varying between countries: *i*) the proportion of fevers in the community that become severe, and *ii*) the proportion of people with SFI that are admitted to inpatient departments. Under the current estimation framework, it was not possible to include information about either of these potential confounders due to lack of available evidence for either across all the countries included in this analysis. For confounder (*i*), the rate at which fevers become severe is highly dependent on the fever’s underlying cause, which is predominantly unknown in community settings, and is was not available at the continuous spatial scale required for inclusion in this analysis. For confounder (*ii*), the treatment-seeking rate for people of all ages for SFI and the subsequent proportion of people who are then admitted was unknown for each country, and therefore not possible to include. Future analyses may seek to quantify these two confounders to reduce noise between datasets from different countries.

Finally, uncomplicated malaria was included in “other causes” of SFI to segregate severe and uncomplicated malaria cases, with the latter often coincident with an undiagnosed NMFI that is the actual underlying cause of the fever [1,8,45]. Thus, separating IPD admissions by severe malaria versus other causes required IPD admission rates to be extracted from modeled estimates produced by Camponovo *et al*. [46], a source with their own uncertain modular inputs.

## Conclusion

This study represents the first estimates of SFI-IPD admissions in SSA. Despite methodological limitations, our findings represent, to our knowledge, the most robust estimates of SFI-IPD admissions available to date. As aptly asserted by Prasad *et al*. (2015) [31], the etiology of many SFIs presenting to IPDs in LMICs remain undiagnosed and thus unknown. This, in turn, may lead to sub-optimal outcomes for patients seeking treatment for SFI. The results presented here highlight again that, in LMICs in SSA and globally, multicenter research is needed to define the major treatable and preventable causes of SFI [3] while efforts to develop, distribute, and sustainably implement multilayered intervention strategies, including reliable routine diagnostics for SFI-causing pathogens and robust data collection mechanisms, should be increased [8]. Our data highlight the enormous scale of the problem, which underlines the need for innovative interventions (i.e. a multiplex multi-analyte diagnostic platform [35]). It further suggests a strong case for investment from funders and developers alike, with a potentially large impact on reducing morbidity and mortality following programmatic implementation.

## Supporting information

S1 Table References for all MoH reports used. (TIFF)
