# Supplementary figures and images for: Quantifying the incidence of severe-febrile-illness hospital admissions in sub-Saharan Africa
Source: PLoS One. 2019 Jul 25;14(7):e0220371. doi: 10.1371/journal.pone.0220371 (PMC6657909; doi:10.1371/journal.pone.0220371)

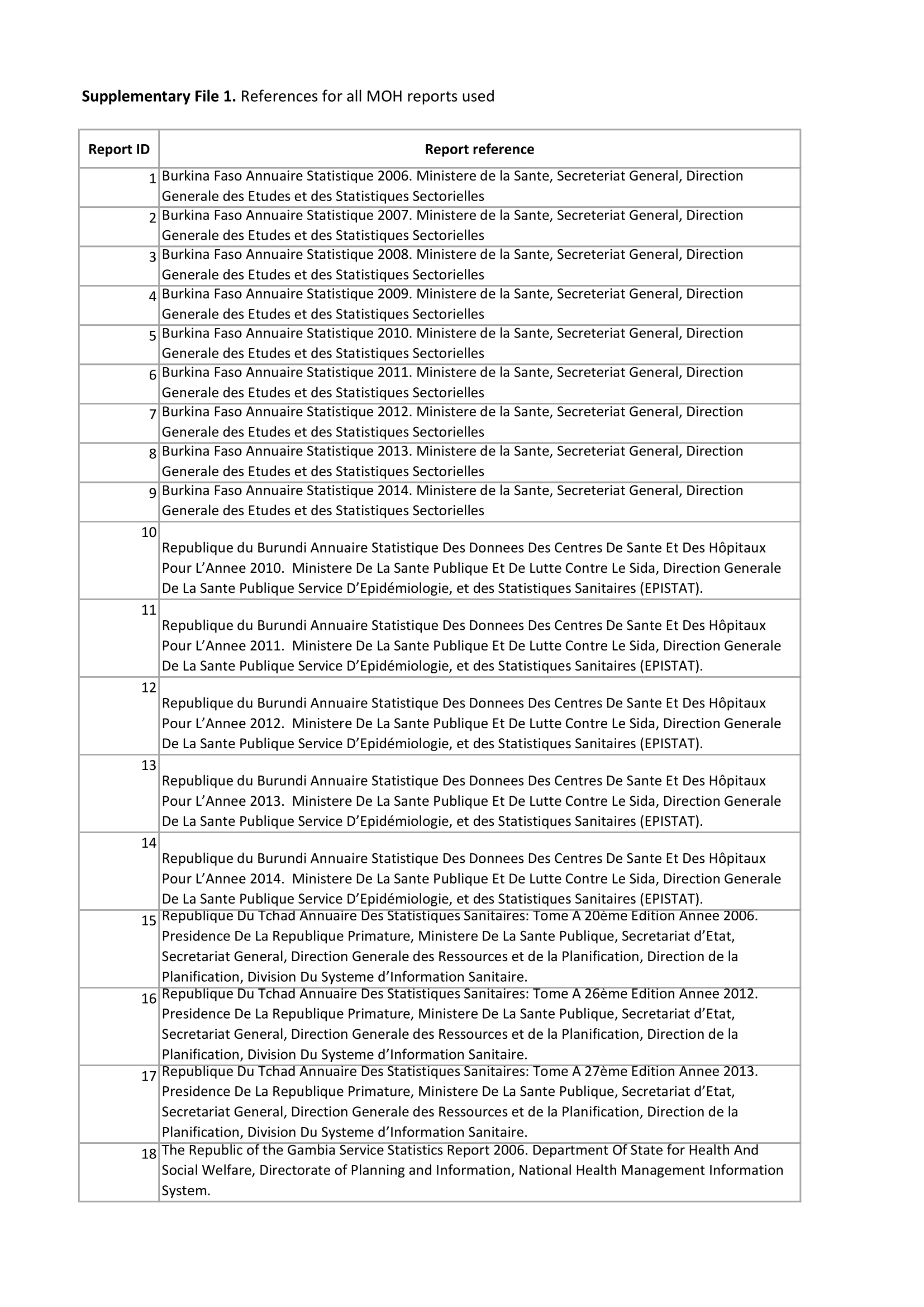

Supplement: S1 Table — (TIFF) [file pone.0220371.s001.tiff]
